# Reactions of industry and associated organisations to the announcement of the UK Soft Drinks Industry Levy: longitudinal thematic analysis of UK media articles, 2016-18

**DOI:** 10.1186/s12889-023-15190-0

**Published:** 2023-02-07

**Authors:** Tarra L. Penney, Catrin P. Jones, David Pell, Steven Cummins, Jean Adams, Hannah Forde, Oliver Mytton, Harry Rutter, Richard Smith, Martin White

**Affiliations:** 1 Global Food System and Policy Research, School of Global Health, Faculty of Health, York University, Toronto, Canada; 2 MRC Epidemiology Unit, University of Cambridge, School of Clinical Medicine, Box 285 Institute of Metabolic Science, Cambridge Biomedical Campus, CB2 0QQ Cambridge, UK; 3 Population Health Innovation Lab, Department of Public Health, Environments & Society, London School of Hygiene & Tropical Medicine, London, UK; 4 Department of Social & Policy Sciences, University of Bath, Bath, UK; 5 College of Medicine and Health, University of Exeter, Exeter, UK

**Keywords:** Fiscal policy, Commercial sector, UK Soft Drinks Industry Levy, Sugar sweetened beverages, Media discourse

## Abstract

**Background:**

The UK Soft Drinks Industry Levy (SDIL) was announced in March 2016, became law in April 2017, and was implemented in April 2018. Empirical analyses of commercial responses have not been undertaken to establish the scale, direction or nuance of industry media messaging around fiscal policies. We aimed to develop a detailed understanding of industry reactions to the SDIL in publicly available media, including whether and how these changed from announcement to implementation.

**Methods:**

We searched Factiva to identify articles related to sugar, soft-drinks, and the SDIL, between 16th March 2016–5^th^ April 2018. Articles included were UK publications written in English and reporting a quotation from an industry actor in response to the SDIL. We used a longitudinal thematic analysis of public statements by the soft-drinks industry that covered their reactions in relation to key policy milestones.

**Results:**

Two hundred and ninety-eight articles were included. After the announcement in March 2016, there was strong opposition to the SDIL. After the public consultation, evolving opposition narratives were seen. After the SDIL became law, reactions reflected a shift to adapting to the SDIL. Following the publication of the final regulations, statements sought to emphasise industry opportunities and ensure the perceived profitability of the soft drinks sector. The most significant change in message (from opposition to adapting to the SDIL) occurred when the SDIL was implemented (6^th^ April 2018).

**Conclusion:**

Reactions to the SDIL changed over time. Industry modified its media responses from a position of strong opposition to one that appeared to focus on adaptation and maximising perceived profitability after the SDIL became law. This shift suggests that the forces that shape industry media responses to fiscal policies do not remain constant but evolve in response to policy characteristics and the stage of the policy process to maximise beneficial framing.

**Supplementary Information:**

The online version contains supplementary material available at 10.1186/s12889-023-15190-0.

## Background

On 16^th^ March 2016, as part of his Spring Budget Statement, the UK Chancellor of the Exchequer announced a new levy on soft drinks to help tackle childhood obesity [1] – the Soft Drinks Industry Levy (SDIL). The SDIL came into effect on 6^th^ April 2018, two years after its announcement, which allowed time for public consultation and for industry to adapt. The SDIL is a levy on companies (rather than consumers directly) that applies to any soft drink with added sugar. It has two-tiers: £0.24/L for drinks containing ≥ 8 g/100ml of added sugar, £0.18/L for drinks containing ≥ 5 g/100ml and < 8 g/100ml. There is no levy on soft drinks containing < 5 g/100ml and exemptions for drinks that are 100% fruit juice, ≥ 75% milk (or milk-replacement), ≥ 1.2% alcohol by volume, alcoholic beverage replacements, or are produced or distributed by manufacturers/importers with < 1 million L/year in UK sales [2]. Regular consumption of sugar-sweetened beverages (SSBs) is associated with total energy intake, and increased incidence of dental caries, obesity, type 2 diabetes and cardiovascular disease [3–6]. In response to these health risks, fiscal dietary policies like the SDIL have been identified as promising tools to reduce consumption [7]. Several SSB taxes of varying designs have been implemented and evaluated internationally [8–13]. These SSB taxes are mainly sales taxes that aim primarily to disincentivise purchases of SSBs. In contrast, the SDIL was designed primarily to incentivise reformulation to reduce the sugar content of drinks.

A reluctance to implement SSB taxes globally can be partially explained by the reactions of the sugary drinks industry to fiscal food policies. Broadly, a range of discursive (i.e. argument based) and instrumental (i.e. action-based) strategies have been used in industry corporate political activity to oppose the imposition of these [14–16]. For example, there have been documented efforts to pre-empt local tax legislation by lobbying municipalities to prohibit levying taxes on foods and beverages in a number of US states [17–20]. In other cases, where local tax bans were not successful, alternative oppositional tactics have been sustained over several years [21, 22]. In Mexico industry funded research was published that casted doubt on whether soda taxes would reduce consumption or improve health, and industry lobbied politicians to vote against SSB taxation [23].

Industry has been reported to make use of media coverage to advocate for industry perspectives, influence societal discourse and policymaking [24–26]. SSBs and fiscal policy more broadly are frequently discussed in the British press, and articles include both public health advocacy and pro-industry messaging [27]. Concerning the SDIL, a media analysis starting a year before the announcement of the policy suggested that articles supportive of SSB taxation (23.5%) outnumbered those that were oppositional (14.2%) [28] and that arguments underpinning proponents and opponents of the SDIL were broadly consistent with those used within the alcohol and tobacco industry [29]. While most of the research examining media representations of industry and their discursive strategies is focused on message framing [30], there is evidence that industry messaging can shift depending on the audience, with conflicting or contradictory debates and responses to regulation [31, 32]. Therefore, examining industry reactions throughout policy development and implementation processes could offer novel insights into how industry messaging evolves in a changing political landscape over time. These insights could inform policy responses to industry attempts to influence the public discourse and subsequently new health policy.

In this paper, we set out to develop a detailed understanding of what approaches were used by industry, and whether and how industry discourse evolved and adapted over time between the announcement and implementation of the SDIL, through qualitative analysis of quotes in the news media and trade press.

## Methods

### Study design

We undertook a longitudinal thematic media analysis from the announcement to the implementation of the Soft Drinks Industry Levy (SDIL).

### Identification and selection of articles

We searched the UK news media (e.g. The Daily Telegraph) and trade press (e.g. The Grocer) in the Factiva database [33]. Searches were conducted in local and global newspapers, newswires, trade journals, newsletters, magazines and transcripts (supplementary file 1) [34]. We sought articles concerning sugar or soft drinks, and the SDIL. As we wanted to capture articles that were primarily about the SDIL, search terms were developed for ‘levy’ and one or more of the following terms: tax*, sugar*, soft drink* and soda* appearing in the title.

All articles were independently screened by two researchers (Researcher 1 and Researcher 3) using predefined inclusion criteria: (a) articles covering the period between the day that the SDIL was announced (16^th^ March 2016) to the day before it was implemented (5^th^ April 2018); (b) articles from UK publications; (c) articles that reported a quotation from an industry, industry-associated think-tank, industry campaign group (e.g. ‘face the facts, can the tax’), or trade body spokesperson in response to the SDIL either from a named individual or non-named spokesperson; and (d) articles published in English (supplementary file 1). Duplicate articles were excluded if they: (a) were a short lead-in to a main story captured in a more in-depth version in the same publication; or (b) were an exact duplicate, for example, articles derived and syndicated from news services such as Reuters.

### Longitudinal analysis

Eligible articles were organised chronologically to allow an analysis that explored potential change in industry response over time (a full list of actors is available on request from the corresponding author). We applied thematic analysis [35] involving six stages: (a) familiarisation; (b) coding; (c) generating initial themes; (d) developing and reviewing themes; (e) refining, defining and naming themes; and (f) writing up. Themes were generated inductively using a data-driven approach as opposed to applying an a priori coding framework informed by previous research or existing theory. This approach was adopted because it allowed us to adopt an exploratory stance appropriate to the context of a novel food tax with a unique structure. Taking a longitudinal and naturalistic perspective of industry media concerning the SDIL enabled qualitative description exploring the who, what, and where of events or experiences [36]. The analysis was not intended to offer a comprehensive account of all industry statements, but to reflect evidence of shifts among important statements that represent the majority of the conversation. No a priori timeline of intermediate events was imposed, allowing for observed shifts in industry responses to be driven by the data. However, the relationship between shifts in messages and events was later identified and documented.

Researcher 1 undertook familiarisation, initial coding and generation of initial themes with input from Researcher 10. Researcher 1, Researcher 2, Researcher 10 & Researcher 4 developed and reviewed the themes while defining and naming themes and the selection of illustrative quotations were derived via consensus with other co-authors. Searching for themes was based on the most salient statements informed by the context of the article. The analysis was aided by Atlas.Ti software [37].

## Results

**Fig. 1:**
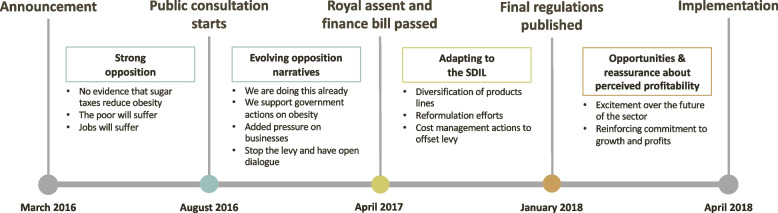
Changing Soft drink industry reactions to the Soft Drinks Industry Levy between announcement and implementation: Themes and sub-themes in relation to key events

Eight hundred and twenty-four articles were screened and 298 met the inclusion criteria (245 news media and 53 trade press from 72 sources). These articles covered the reactions of the soft drinks industry, industry associations, industry movements (e.g. ‘Can the Tax’) and think-tanks. Table 1 describes the key policy development milestones derived inductively from this analysis and Table 2 describes the number of eligible articles found for each time period. The temporal sequencing of the relative salience of these themes are shown in relation to these milestones. Five themes reflecting industry reactions to the SDIL emerged from our analysis; these are summarised in Fig. 1.

**Table 1:** Description of key policy process milestones of the SDIL in the United Kingdom, derived inductively from the longitudinal thematic analysis

**Policy announcement:** On 16^th^ March 2016, Chancellor George Osborne announced the introduction of the Soft Drinks Industry Levy in his Budget Speech to Parliament. In his announcement he stated that the SDIL would be levied on companies and introduced in 2 years (April 2018) to allow companies time to “change their product mix” [38].
**Public consultation:** On 18^th^ August 2016, HM Revenue and Customs and HM Treasury published details of a public consultation on the SDIL. The aim of this public consultation was to capture views on the impact of the proposed SDIL and its particulars. Organisations and individuals were invited to read and comment on the proposals by 13^th^ October 2016 [39].
**Royal assent and passing of finance bill:** On 25^th^ April 2017, Parliament debated and passed the Finance Bill which pertained to the SDIL [40]. Once a bill has passed through Parliament it receives Royal Assent, whereby Her Majesty The Queen formally agrees to pass the bill into law [41].
**Publication of final regulations:** HM Revenue and Customs published the final regulations for the SDIL on 15^th^ January 2018. These were laid before the House of Commons on 17^th^ January 2018 and detail the final particulars of the SDIL [42].
**Implementation:** The SDIL came into effect on 6^th^ April 2018, with a levy placed on sugar sweetened beverages from this date.

**Table 2:** Number of articles included in the analysis from each time period

Time period	Dates	Number of articles
Announcement of SDIL – Public Consultation	16th March 2016–17th August 2016	175
Public Consultation – Royal Assent & Passing of Finance Bill	18th August 2016–24th April 2017	73
Royal Assent & Passing of Finance Bill – Publication of Final Regulations	25th April 2017–14th January 2018	30
Publication of Final Regulations – Implementation of SDIL	15th January 2018–5th April 2018	19

After the announcement on 16^th^ March 2016, there was strong opposition. Following the public consultation (18^th^ August-13^th^ October 2016), reactions demonstrated continued opposition but with important shifts in the narrative. After the finance bill was passed and received Royal Assent (i.e. the SDIL became UK law) on 27^th^ April 2017, reactions reflected adapting to the SDIL. Following the publication of the final regulations on 15^th^ January 2018, and as the date of implementation approached (6^th^ April 2018), industry statements sought to demonstrate opportunities and reassurance concerning perceived profitability of the soft drinks sector. Overall, reactions were consistent across different industry actors in each of these time periods. The most significant change in message (from opposition to tolerance and reassurance concerning the impact of the SDIL on industry) occurred after the passing of the Finance Bill.

### Prior to the SDIL becoming UK Law (March 2016 to April 2017)

Immediately after the announcement of the SDIL, as part of the March 2016 budget and before the SDIL become UK law, industry reacted with strong opposition to the introduction of the SDIL.

#### Strong opposition

The initial oppositional stance comprised multiple dimensions, primarily with statements questioning the evidence base underlying the use of SSB taxes to support behaviour change and reductions in obesity. For example, Coca-Cola European Partners Vice President and General Manager, said: “We don’t believe the sugar tax is the right thing to be done. We are not debating the issue, we are debating the solution. The facts don’t suggest that a sugar tax works to change behaviour.” (The Guardian, 17^th^ March 2016). The chief executive of Nichols, said: “While we recognise sugar consumption is a shared responsibility, we do not believe that a tax on soft drinks is an effective solution or fair to consumers.” (The Guardian, 17^th^ March 2016). Statements questioning the underlying evidence were accompanied by claims that the SDIL would increase costs for the public, regardless of what they drink. For example, the General Manager of Coca-Cola UK & Ireland said: “It will also hit consumers in the pocket regardless of what they drink.” (The Daily Telegraph, 26^th^ May 2016). Industry also discussed the progress already made in reducing sugar, and suggested SSBs were unfairly targeted; AG Barr chief executive stated: “It is extremely disappointing that soft drinks have been singled out given it is the only food and drink category to have made any real progress in reducing sugar intake in recent years, down 13.6 per cent since 2012.” (Daily Record.co.uk, 17^th^ March, 2016).

Industry associations also made statements on behalf of their membership and the industry more broadly. For example, Food and Drink Federation Director General, said: “For nearly a year we have waited for an holistic strategy to tackle obesity. What we’ve got today instead is a piece of political theatre. … The imposition of this tax will, sadly, result in less innovation and product reformulation, and for some manufacturers is certain to cost jobs. Nor will it make a difference to obesity.” (Daily Mirror Online, 17^th^ March, 2016) In addition, a spokesperson for the pro-industry think-tank, the Institute of Economic Affairs, said: “It is astonishing that the Chancellor has announced a tax on sugary drinks when there is no evidence from anywhere in the world that such taxes have the slightest effect on obesity.” … “Whether dressed up as a direct tax or a levy on industry, the effect will be that the Government will be picking the pockets of the poor for no benefit.” (Mail Online, 17^th^ March 2016) During this time, there was also one mention of the potential for legal action by the soft drinks industry, reported as from a “senior industry source”: “It’s fair to say we are more than just considering legal action. This has been rushed through without warning”. (The Sunday Times, 20th March, 2016)

#### Evolving opposition narratives

In the months leading up to the launch of the public consultation in August 2016 and until the consultation closed in October 2016, the immediate and primarily strong oppositional stance continued. Industry continued to highlight the impact on jobs and added pressure on their business, continuing to question the legislation, with a desire to have an open dialogue between government and industry. For example, a Coca-Cola spokesman said: “Coca-Cola works with governments and civil society around the world on calorie reduction initiatives that we believe are more effective than discriminatory taxes. … We believe it is important that industry and government keep an open and constructive dialogue on this issue.” (The Sunday Times, 16^th^ October, 2016). The British Soft Drinks Association Director General, said: “Given current increases in the cost of goods, we’re surprised the Treasury wishes to put more pressure on businesses and raise prices for hard-pressed consumers.” … “It’s also ironic that the tax hits the soft drinks category, which has led the way in helping consumers reduce sugar intake - down nearly 18% since 2012. We are also the only sector with a calorie reduction target for 2020.” … “We support the need to address the public health challenge the country faces, but it’s worth bearing in mind that there is no evidence taxing a single product or ingredient has reduced levels of obesity anywhere in the world.” (Mail Online, 9^th^ March, 2017).

The narrative also subtly shifted and increasingly highlighted existing commitments of the soft drinks industry, such as voluntary actions supporting government public health policies on sugar and calorie reduction and obesity. AG Barr’s chief executive, said “Our job is to understand and have relationships with our customers, which we have had for over 100 years, making sure we offer them choices. If they want standard Irn-Bru they can have that just as they do at the moment,” … “I would continue to be disappointed that despite the considerable voluntary initiatives and the significant progress that our industry and the company have made, a punitive levy on a specific single category has been brought out where, in stark contrast to other food and drink categories, we have been reducing sugar content and have a strong [commitment] to do so.” (The Guardian, 6^th^ May, 2016). The Chief Executive of Britvic said that two-thirds of the group’s UK portfolio already fell outside the tax: “Britvic already offers a broad range of products and has taken significant steps in recent years to reduce added sugar and reformulate drinks, using Stevia for example, and remains committed to a 20 per cent calorie reduction by 2020.” (Financial Times, 19^th^ May, 2016).

### After the SDIL became UK Law (April 2017 to April 2018)

In the immediate lead up to, and after, the SDIL became UK law on 27^th^ April 2017, the industry narrative shifted to one of adaptation to the SDIL, outlining strategies in reaction to potential financial impacts, and statements about ensuring perceived profitability.

#### Adapting to the SDIL

While the underlying opposition remained, statements were more focused on explaining their company specific strategies for adapting to the SDIL, particularly to preserve profits. These included reformulation efforts, diversification and other actions that would be used to offset the cost of the SDIL to manufacturers. Lucozade Ribena Suntory’s Director of Research and Development, said: “We are taking out 50% of sugar this year across the whole portfolio and that actually means that for each drink they’ll be 4.5 g of sugar per 100ml or less…So we are going to make sure for each brand that we have a zero calorie or no added sugar variant. That’s a massive change for us, but we also recognise that that is not enough, so we are actually committing £30 M of investment across the next three years to get more people exercising and starting that with our own employees.” (Food Manufacture, 1st June 2017). Coca-Cola European Partners Marketing Director said: “For those who like the taste of Coke but want zero sugar we have Coke Zero Sugar, which is now the fastest-growing cola in Britain. We’re already seeing a shift in mix.” (The Grocer, 22^nd^ April 2017).

Similarly, Britvic’s Chief Executive made various statements indicating that while he admitted the SDIL would bring uncertainty to the industry, they were prepared. For example, he said: “94% of our own brands will be unaffected by the sugar levy. Pepsi and 7UP are the prime brands that can be affected but they also have low sugar offers”. He also said that the company raised prices to offset the impact of a weaker pound and higher raw material costs, and added that the company also reduced costs by £8 million ($10.7 million) in the year (Reuters News, 29^th^ November 2017). Chief Financial Officer for Vimto (owned by Nichols plc), made a statement about a recipe reformulation project that would mean none of its products would be vulnerable to the SDIL. They said: “Last year, 70pc of our UK growth came from no added sugar and zero variants,” … “It has also been helpful that the acquisitions [of Noisy Drinks and Feel Good] have produced drinks which are below the levy.” (The Telegraph Online, 21^st^ July 2017).

#### Opportunities and reassurance about perceived profitability

After the final regulations had been published on 15^th^ January 2018, and as the implementation date for the SDIL approached on 6^th^ April 2018, media statements appeared to be aimed at reassuring the public and shareholders about the perceived profitability of the soft drinks sector, and excitement about the future. However, underlying opposition remained. For example, Britvic’s Chief Executive, said: “We have delivered a solid start to the new financial year, with group revenue growing 3.3% ahead of a strong first quarter last year. As we said at our preliminary results, the introduction of a Soft Drinks Industry Levy in the UK and Ireland brings a level of uncertainty, but we are well placed to navigate this given the strength and breadth of our brand portfolio and exciting marketing and innovation plans.” Additional statements were about how the combination of cost management and progress will ensure a bright future. “In addition, our continued focus on revenue and cost management and the delivery of the final phase of our business capability programme means we remain confident of making further progress in 2018.” (Mail Online, 31^st^ January 2018) While statements around profitability continued, additional statements regarding future opportunities for the business were also highlighted. For example, referring to new product launches in the UK the General Manager of Coca-Cola for Britain and Ireland said: “These launches have moved much quicker through the business than before, this is a sign of things to come.” (The Telegraph Online, 29^th^ January 2018).

## Discussion

### Summary of main findings

The purpose of this analysis was to examine in what ways the soft drinks industry reacted to the SDIL in the public media, and how this industry narrative changed over a two-year period spanning the development and implementation of the policy. Our findings suggest that soft drinks industry sentiment in the news and trade media was orchestrated across the industry and evolved over time, with a significant shift in prevailing sentiment once the SDIL had achieved Royal Assent in April 2017. Before this, industry demonstrated strong opposition and advocated for slowing or halting the policy. Arguments centred on the economic impact of the policy, suggesting that it would undermine industry, cost jobs and threaten the UK economy, as well as a prevailing narrative that it would not be effective for public health. Post-April 2017, sentiment shifted to focus on commercial strategies that could counter the potential financial impact of the SDIL and ensure continued profitability in the sector.

### Strengths and limitations

A strength of this analysis is the longitudinal approach, which permits an examination of how industry messaging about the SDIL evolved over time. Previous analyses of media responses to the SDIL span from 11 months pre-announcement to 8 months post-announcement and used content and discourse network analyses to explore sentiment around the SDIL announcement [28, 29, 43]. Our analysis complements and extends this work by examining industry media sentiment until the implementation of the SDIL in April 2018, as well as using thematic analysis inductively to explore how this changed throughout this period. Media data is curated by journalists, editors and actors but our focus on specific statements made by industry actors sought to reduce the impact of editorial content and achieve greater fidelity than by analysing entire articles. By focusing on these direct statements, we were able to attain insights into industry reactions to the SDIL that they wished to make public. To ensure that the broadest range of industry views was reflected, we also included quotes from industry associations, industry movements and industry-associated think-tanks, as well as quotes in the trade press. Including trade press gives unique insights into industry conversations with competitors and shareholders, as well as the more public-facing messaging to shape public opinion and policy debates in news media. Across media sources, statements by industry may reflect a wider communication strategy to position themselves in a particular way in the eyes of policymakers/potential consumers, rather than reflecting the true positions, values or behaviours of either individuals or organisations with regard to SSB taxes.

There are some limitations to this work however. Although we aimed to explore industry reaction to the policy process through the news media (i.e. how they sought to influence policymaking through this medium), we did not explore industry reaction once the policy was implemented. Further, whilst comprehensive searches were conducted in news and trade press, this work does not include industry statements given through other forms of communication, for example social media. Finally, this work takes a higher-level position exploring ‘industry’ reaction as a whole and there may be some nuance missed by approaching the research in this way.

### Relationship to prior knowledge

News media provides industry with a platform to present messages favourable to their strategy. Some of these messages given in response to unfavourable policy, have been described as an industry ‘playbook’ of typical responses, which primarily centre around complexity arguments as well as framing evidence-using techniques such as ‘siloing’ (the separation of contradictory arguments) or ‘boomeranging’ (the weaponisation of logic from their opponents) [44, 45]. Our findings suggest that the industry ‘playbook’ was used prior to the SDIL becoming law; however, once the SDIL was certain to be introduced, media messaging started to transition away from this playbook, and sought to emphasise that they are not only adapting to the SDIL but it could be used as an opportunity to increase profit. Alternatively, as opposed to a transition away from the playbook, our research could highlight an area that has been underexplored previously: reassuring shareholders that they will still be profitable following new fiscal policy. Exploration of news media in South Africa following the announcement of plans to introduce an SSB tax identified less negative media coverage than we found in the UK in relation to the SDIL [46]. It could be that an alternative playbook strategy to ‘strong opposition’ is ‘reassurance of profitability’, but this was not the initial preferred response to the UK SDIL by industry representatives speaking to the news media.

Following the implementation of the SDIL, evidence suggests that these concerns from the ‘industry playbook’ did not materialise. Although a negative impact on domestic turnover occurred in the short-term, this was not sustained following implementation in April 2018 [47], and although there was an negative stock market reaction immediately after the announcement of the SDIL (for the four UK soft drink firms listed on the London Stock Exchange), after four days this effect had dissipated [48]. Evidence examining SSB taxation internationally corroborates these findings, that little negative economic impact occurs following implementation [49]. Early industry claims of adverse economic impacts and job losses, therefore, did not occur.

### Interpretation and implications for policy and practice

Overall, articles suggested that industry actors took a strategic approach to monitoring and change. Although this approach showed similarities with previous work exploring the ‘industry playbook’, the design of the SDIL was somewhat different from the majority of previous SSB taxes, which tended to be direct taxes on sales, and thus led directly to price increases for consumers. The SDIL is levied on manufacturers and importers in two tiers, according to the sugar concentration of drinks, and is intended to incentivise industry to reformulate SSBs [42]. In contrast to a tax designed only to reduce demand for SSBs via increased product price, this approach provides an opportunity for industry to avoid the paying the levy by reformulating their drinks. Findings exploring industry responses to the SDIL from 2015 to 2019 support this and show a large reduction in sugar content in eligible drinks, demonstrating that many companies were able to avoid paying the levy [50]. The shift in sentiment from one of opposition to one of adaptation and ensuring perceived profitability could be attributed to this response – that industry were able to avoid the SDIL unlike SSB taxes implemented elsewhere. However, this shift in sentiment also occurred after the SDIL was given Royal Assent and became law. Industry appears to have collectively changed their narrative around the SDIL once it was unavoidable, as public oppositional stances could no longer prevent its implementation after April 2017. The stance adopted once the SDIL became inevitable (from April 2017), in contrast, appeared to be aimed at reassuring shareholders concerning profitability. This work highlights that strong industry opposition to policies prior to them becoming law may not reflect real threats to industry but merely be tactical expressions aimed at hindering or modifying policy that could impact their profits and operations. Policymakers designing food taxes, or other regulatory food policies, need to be aware of industry media responses, including how these typically adhere to a standard ‘playbook’ of oppositional tactics. Alongside the shift in content we describe, this change in volume could indicate a shift in the industry prioritisation of resources to responding internally and adapting to the policy, rather than commenting externally. Policymakers should also be aware, therefore, that oppositional responses may reduce in volume once a policy is made law and is inevitable.

### Unanswered questions and future research

Similar to work conducted on the UK Public Health Responsibility Deal [51, 52], future research could use Freedom of Information (FOI) requests to explore food industry communication regarding the SDIL. This would allow insights to be gained into the non-public positions of industry regarding the SDIL and the relationship of these positions could be compared to the findings from this research. Comparisons could also be made between industry reactions to the SDIL through the news media or FOI requests and similar responses to other health policies, for example for tobacco or alcohol control. This would allow researchers to explore whether the patterns found in this paper represent a standard industry playbook, or whether they express real adaptations and learning by industry in response to the SDIL specifically. Future research could explore industry reaction through quotes given to the news media following the implementation of the SDIL in April 2018, as well as exploring in more detail the differences between industry actors, types of press (general news vs. trade press), and also examine the reaction expressed via industry press releases or industry umbrella groups.

## Supplementary Information

**Additional file 1: Supplementary File 1.** Number of included articles per media publication, categorised by trade and news media. **Supplementary File 2.** List of news media articles included in the analysis. **Supplementary File 3.** Full List of actors and the frequency of inclusion in included articles.

## Data Availability

The datasets used and/or analysed during the current study are available as supplementary materials.
