# Supplementary material for: Reactions of industry and associated organisations to the announcement of the UK Soft Drinks Industry Levy: longitudinal thematic analysis of UK media articles, 2016-18
Source: BMC Public Health. 2023 Feb 7;23:280. doi: 10.1186/s12889-023-15190-0 (PMC9906897; doi:10.1186/s12889-023-15190-0)
Supplement: Supplementary file 1 — Additional file 1: Supplementary File 1. Number of included articles per media publication, categorised by trade and news media. Supplementary File 2. List of news media articles included in the analysis. Supplementary File 3. Full List of actors and the frequency of inclusion in included articles. [file 12889_2023_15190_MOESM1_ESM.docx]

Supplementary File 1: Number of included articles per media publication, categorised by trade and news media.

| **Media Type** | **Publication** | **Number of Articles** |
| --- | --- | --- |
| News Media | Barry And District News | 1 |
| News Media | Bath Chronicle | 1 |
| News Media | Belfast Telegraph | 3 |
| News Media | Bournemouth Echo | 2 |
| News Media | Bradford Telegraph and Argus | 1 |
| News Media | Bristol Evening Post | 1 |
| Trade Press | British Baker | 2 |
| News Media | Bury Free Press | 1 |
| News Media | Carmarthen Journal | 1 |
| News Media | City AM | 8 |
| News Media | Citywire | 1 |
| News Media | Coventry Telegraph | 1 |
| News Media | Daily Record | 6 |
| News Media | Daily Star | 1 |
| News Media | dailystar.co.uk | 1 |
| News Media | Dorset Echo | 1 |
| News Media | Eastern Daily Press | 3 |
| News Media | ENP Newswire | 1 |
| News Media | Evening Express | 1 |
| News Media | Evening News (Norwich) | 2 |
| News Media | Evening Times | 1 |
| News Media | express.co.uk | 4 |
| News Media | Financial Times | 8 |
| News Media | Financial Times (FT.Com) | 5 |
| Trade Press | FinancialWire | 1 |
| Trade Press | Food Manufacture | 3 |
| News Media | Grantham Journal | 1 |
| News Media | Huddersfield Examiner | 1 |
| News Media | i | 4 |
| News Media | Independent Online | 10 |
| Trade Press | Investors Chronicle - Magazine and Web Content | 1 |
| News Media | irishmirror.ie | 1 |
| Trade Press | Just-Drinks | 20 |
| Trade Press | Just-Food | 3 |
| News Media | Knutsford Guardian | 1 |
| News Media | Lakeland Echo | 1 |
| News Media | London Evening Standard | 3 |
| News Media | London Evening Standard Online | 5 |
| News Media | Mail Online | 15 |
| Trade Press | Marketing | 1 |
| News Media | Milton Keynes Citizen | 1 |
| News Media | Mirror.co.uk | 2 |
| News Media | Oxford Mail | 1 |
| Trade Press | Reuters Health E-Line | 1 |
| News Media | Reuters News | 5 |
| News Media | Scottish Daily Mail | 8 |
| News Media | Somerset County Gazette | 1 |
| News Media | Tamworth Herald | 1 |
| News Media | Telegraph Magazine | 1 |
| News Media | The Daily Express | 5 |
| News Media | The Daily Mirror | 2 |
| News Media | The Daily Telegraph | 20 |
| Trade Press | The Grocer | 19 |
| News Media | The Guardian | 16 |
| News Media | The Herald | 8 |
| News Media | The Independent | 4 |
| News Media | The Irish Times | 1 |
| News Media | The Journal, Newcastle | 2 |
| News Media | The Press and Journal | 1 |
| News Media | The Sun | 4 |
| News Media | The Sunday Telegraph | 2 |
| News Media | The Sunday Times | 5 |
| News Media | The Sunderland Echo | 1 |
| News Media | The Telegraph Online | 16 |
| News Media | The Times | 10 |
| News Media | The Western Mail | 2 |
| Trade Press | Theflyonthewall.com | 1 |
| News Media | thescottishsun.co.uk | 5 |
| News Media | thesun.co.uk | 21 |
| News Media | thetimes.co.uk | 4 |
| News Media | Western Daily Press | 1 |
| News Media | Wiltshire Times | 1 |

Supplementary File 2: List of news media articles included in the analysis.

| **Article Title** | **Day** | **Month** | **Year** | **Actor Category** | **All Industry Actors** | **Type of Publication** | **Publication** |
| --- | --- | --- | --- | --- | --- | --- | --- |
| UK unveils sugar tax on soft drinks, freezes beer duty. | 16 | 3 | 2016 | Association | British soft drinks association | Trade Press | Just-Drinks |
| Osborne's ludicrous sugar tax didn't sweeten a bland Budget. | 17 | 3 | 2016 | Think-Tank | Institute of economic affairs | News Media | City AM |
| Bitter news for Irn Bru makers AG Barr as budget sugar tax causes shares to divebomb. | 17 | 3 | 2016 | Drinks Industry | AG Barr, Food and drink federation | News Media | dailyrecord.co.uk |
| Shares in AG Barr drop 5% on Chancellor's sugar tax announcement. | 17 | 3 | 2016 | Drinks Industry | AG Barr | News Media | dailyrecord.co.uk |
| Surprise sugar tax will be used to halt rise in childhood obesity. | 17 | 3 | 2016 | Association | Food and drink federation / Tax payers alliance | News Media | Eastern Daily Press |
| Chancellor introduces sugar tax. | 17 | 3 | 2016 | Association | Food and drink federation | News Media | Evening News (Norwich) |
| Budget 2016: What is the sugar tax on soft drinks? | 17 | 3 | 2016 | Association | British soft drinks association, food and drink federation | News Media | express.co.uk |
| Makers of Irn-Bru dismayed by sugar tax. | 17 | 3 | 2016 | Drinks Industry | AG Barr | News Media | express.co.uk |
| Budget 2016: George Osborne's sugar tax hides £55bn black hole in public finances. | 17 | 3 | 2016 | Association | Food and drink federation | News Media | Independent Online |
| Will the sugar tax stop children having fizzy drinks and will it really make Britain less fat? | 17 | 3 | 2016 | Association | British soft drinks association | News Media | Independent Online |
| Which drinks are affected by the sugar tax? | 17 | 3 | 2016 | Drinks Industry | AG Barr | News Media | Lakeland Echo |
| Budget 2016: Sugar tax on sweetened drinks announced. | 17 | 3 | 2016 | Drinks Industry | AG Barr | News Media | London Evening Standard Online |
| Osborne's sugar tax hits firms with £500m bill: Shares plummet after announcement of levy that could add 25p to cost of Coca Cola and Red Bull. | 17 | 3 | 2016 | Drinks Industry | Coca-Cola | News Media | Mail Online |
| Osborne's sugar tax leaves bitter taste: Soft drinks crackdown to fight childhood obesity is branded a 'fat tax' with 'no evidence of success. | 17 | 3 | 2016 | Think-Tank | Institute of economic affairs, Adam Smith Institute, Taxpayers alliance, British soft drinks association, AG Barr, Food and drink federation | News Media | Mail Online |
| Sugar tax in the Budget could add 24p a litre to a bottle of Coca Cola. | 17 | 3 | 2016 | Association | Food and drink federation | News Media | Mirror.co.uk |
| Osborne's sugar tax shock hits fizzy drink firms with £500m bill. | 17 | 3 | 2016 | Drinks Industry | AG Barr, Scottish grocers federation | News Media | Scottish Daily Mail |
| Drinks giants' fury over tax on sugar. | 17 | 3 | 2016 | Association | British soft drinks association | News Media | Scottish Daily Mail |
| Jamie jubilant over Weather forecast shock sugar tax on soft drinks. | 17 | 3 | 2016 | Association | British soft drinks association, food and drink federation | News Media | The Daily Express |
| Jamie jubilant over shock sugar tax on soft drinks. | 17 | 3 | 2016 | Association | British soft drinks association | News Media | The Daily Express |
| Barr's dismayed over sugar tax. | 17 | 3 | 2016 | Drinks Industry | AG Barr | News Media | The Daily Express |
| Sugar tax causes a stir in battle against childhood obesity. | 17 | 3 | 2016 | Citizen | Tax payers alliance | News Media | The Daily Telegraph |
| Sugar tax: Osborne's two-tier levy brings mixed response. | 17 | 3 | 2016 | Association | British soft drinks association, Food and drink federation | News Media | The Guardian |
| Sugar tax hides the bitter truth of Treasury's £55bn black hole. | 17 | 3 | 2016 | Association | British soft drinks association, Food and drink federation | News Media | The Independent |
| TV chef welcomes sugar tax as 'profound move'. | 17 | 3 | 2016 | Association | Tax payers alliance, British soft drinks association, AG Barr, Food and drink federation | News Media | The Journal, Newcastle |
| Irn Bru-ised - fizzy drinks shares hit by new sugar tax. | 17 | 3 | 2016 | Drinks Industry | AG Barr, CBI | News Media | The Press and Journal |
| Soft drinks makers fall flat after sugar tax surprise. | 17 | 3 | 2016 | Association | Associated British foods | News Media | The Times |
| Irn Brute: Budget bombshell as George Osborne slaps sugar tax on soft drinks. | 17 | 3 | 2016 | Drinks Industry | AG Barr, Scottish grocers federation. | News Media | thescottishsun.co.uk |
| Irn Brute: George Osborne slaps sugar tax on soft drinks. | 17 | 3 | 2016 | Drinks Industry | AG Barr, Scottish grocers federation | News Media | thesun.co.uk |
| Osborne announces sugar tax as he unveils spending cuts. | 17 | 3 | 2016 | Citizen | Tax payers alliance | News Media | thetimes.co.uk |
| Sugar tax is welcomed by health chiefs. | 17 | 3 | 2016 | Citizen | Tax payers alliance | News Media | Western Daily Press |
| Sugar tax is a positive step in helping to tackle health issues such as diabetes and obesity. | 18 | 3 | 2016 | Drinks Industry | Princes | News Media | Bradford Telegraph and Argus |
| Job loss warning over sugar tax. | 18 | 3 | 2016 | Association | Food and drink federation | News Media | Coventry Telegraph |
| No rebellion Bru-ing on sugar tax. | 18 | 3 | 2016 | Drinks Industry | AG Barr | News Media | Daily Record |
| Future less sweet for soft drinks groups; Budget: Sugar tax. | 18 | 3 | 2016 | Association | British soft drinks association, Coca-Cola | News Media | Financial Times |
| Coca-Cola says sugar tax will not reduce childhood obesity. | 18 | 3 | 2016 | Drinks Industry | Coca-Cola | News Media | Independent Online |
| The sugar tax backlash: Osborne's new levy could force up price of diet drinks and even bottles of water, say critics. | 18 | 3 | 2016 | Think-Tank | Institute of economic affairs, Coca-Cola, Adam Smith institute, British soft drinks association, Tax payers alliance | News Media | Mail Online |
| Sugar levy could push up price of bottled water. | 18 | 3 | 2016 | Think-Tank | Institute of economic affairs | News Media | Scottish Daily Mail |
| Osborne's sugar tax is a money-making gimmick that will do nothing to combat child obesity. | 18 | 3 | 2016 | Association | Automatic vending association | News Media | The Daily Telegraph |
| From sugar tax to big data: 9 lessons about tackling obesity. | 18 | 3 | 2016 | Association | British retail consortium | News Media | The Guardian |
| Coca-Cola and other soft drinks firms hit back at sugar tax plan. | 18 | 3 | 2016 | Drinks Industry | Coca-Cola, Nichols, Britvic | News Media | The Guardian |
| Letters: Osborne’s sugar tax is a money-making gimmick that will do nothing to combat child obesity. | 18 | 3 | 2016 | Association | Automatic vending association | News Media | The Telegraph Online |
| Swinney wants details of sugar tax impact. | 18 | 3 | 2016 | Drinks Industry | AG Barr, Scottish grocers federation | News Media | The Times |
| What could surprise UK sugar drinks tax mean for food industry? | 19 | 3 | 2016 | Association | Food and drink federation | Trade | Just-Food |
| Soft drinks industry reels over scale of Osborne's sugar tax. | 19 | 3 | 2016 | Association | Food and drink federation | Trade | The Grocer |
| Sunday Papers: Soft drink giants prepare to sue over sugar tax. | 20 | 3 | 2016 | Drinks Industry | Coca-Cola | News Media | Citywire |
| Fat chance that sugar tax is the answer, Jamie. | 20 | 3 | 2016 | Drinks Industry | AG Barr | News Media | The Sunday Times |
| Soft drink giants prepare to sue over sugar tax. | 20 | 3 | 2016 | Drinks Industry | Coca-Cola | News Media | The Sunday Times |
| Sugar tax: soft drinks makers including Coca-Cola consider suing the Government. | 21 | 3 | 2016 | Drinks Industry | Coca-Cola, British soft drinks association | News Media | Independent Online |
| Drinks giant in legal war on sugar tax: Coca-Cola and Britvic among manufacturers planning legal challenge over claims that the new laws are unfair. | 21 | 3 | 2016 | Association | British soft drinks association, SOURCES - Anonymous | News Media | Mail Online |
| Drinks giants in legal war on sugar tax. | 21 | 3 | 2016 | Association | British soft drinks association, SOURCES - Anonymous | News Media | Scottish Daily Mail |
| Fizzy drinks firms to sue over sugar tax plan. | 21 | 3 | 2016 | Drinks Industry | SOURCE - Anonymous | News Media | The Daily Express |
| Osborne faces legal action on sugar tax. | 21 | 3 | 2016 | Drinks Industry | Coca-Cola, Britvic, SOURCE - Anonymous | News Media | The Daily Mirror |
| Soft-drinks makers consider legal action as they warn sugar tax could jeopardise sector. | 21 | 3 | 2016 | Association | British soft drinks association | News Media | The Daily Telegraph |
| Drinks makers consider legal action against sugar tax. | 21 | 3 | 2016 | Association | British soft drinks association, Coca-Cola | News Media | The Guardian |
| Sugar tax will ‘push the poor further into poverty’. | 21 | 3 | 2016 | Drinks Industry | AG Barr | News Media | The Herald |
| Will the sugar tax leave marketeers with a crashing low? | 22 | 3 | 2016 | Think-Tank | Advertising association, Institute of promotional marketing, Lick creative, Incorporated Society of British Advertisers | News Media | City AM |
| What does McDonald's think about sugar taxes? | 22 | 3 | 2016 | Food Industry | McDonald's | Trade | Just-Food |
| Cooler heads will prevail on UK sugar tax - Coca-Cola Enterprises CEO. | 23 | 3 | 2016 | Drinks Industry | Coca-Cola enterprises | Trade | Just-Drinks |
| Who will be hit hardest by UK sugar tax? - Analysis. | 23 | 3 | 2016 | Food Industry | Tesco, Coca-Cola, | Trade | Just-Drinks |
| Hidden £1bn sugar tax cost. | 23 | 3 | 2016 | Citizen | People against sugar tax | News Media | Scottish Daily Mail |
| Chancellor's sugar tax plan will be watered down in yet another Budget U-turn, Coca Cola exec claims. | 23 | 3 | 2016 | Drinks Industry | Coca-Cola, British soft drinks association | News Media | thesun.co.uk |
| 'Bring it on!' Sugar tax will be 'robustly' defended in the courts says George Osborne as he warns drinks companies not to 'waste time and money on a legal challenge'. | 24 | 3 | 2016 | Drinks Industry | Coca-Cola, AG Barr, Britvic, British soft drinks association | News Media | Mail Online |
| 'Bring it on': George Osborne ready for legal battle with Coca-Cola over sugar tax. | 25 | 3 | 2016 | Drinks Industry | Coca-Cola | News Media | thesun.co.uk |
| AG Barr profits ‘to lose fizz’ after sugar tax blow. | 26 | 3 | 2016 | Drinks Industry | AG Barr | News Media | express.co.uk |
| We're already below the sugar tax threshold. | 26 | 3 | 2016 | Drinks Industry | Apply Food & Drinks | Trade | The Grocer |
| How the sugar tax will hit soft drinks pricing. | 26 | 3 | 2016 | Drinks Industry | Britvic, Coca-Cola enterprises | Trade | The Grocer |
| Soft drinks left carrying the can with sugar levy. | 26 | 3 | 2016 | Drinks Industry | British soft drinks association, Coca-Cola enterprises | Trade | The Grocer |
| Sweet milkshakes escape sugar tax. | 27 | 3 | 2016 | Food Industry | McDonald's | News Media | The Sunday Times |
| Barr's not so sweet on Chancellor's 'sugar tax'. | 28 | 3 | 2016 | Drinks Industry | AG Barr | News Media | The Journal, Newcastle |
| Sugar tax may leave drinks firm a little flat. | 28 | 3 | 2016 | Drinks Industry | AG Barr | News Media | The Western Mail |
| UPDATE 2-AG Barr to cut sugar in soft drinks ahead of UK levy. | 29 | 3 | 2016 | Drinks Industry | AG Barr | News Media | Reuters News |
| Sugar tax battle for soft drinks maker. | 29 | 3 | 2016 | Drinks Industry | AG Barr | News Media | Scottish Daily Mail |
| Irn Bru maker to shun high-sugar drinks over new tax. | 30 | 3 | 2016 | Drinks Industry | AG Barr | News Media | Belfast Telegraph |
| Sugar tax won’t hit us - and the future’s looking really healthy. | 30 | 3 | 2016 | Drinks Industry | More Drinks | News Media | Bristol Evening Post |
| A spoonful of sugar tax can help fizzy drinks go down. | 30 | 3 | 2016 | Drinks Industry | AG Barr | News Media | City AM |
| Tories' sugar tax could be about to force a change in top-secret recipe. | 30 | 3 | 2016 | Drinks Industry | AG Barr | News Media | Daily Record |
| Sugar tax is a bitter draught for drinks maker AG Barr to swallow. | 30 | 3 | 2016 | Drinks Industry | AG Barr | News Media | Financial Times |
| Sugar tax sours AG Barr. | 30 | 3 | 2016 | Drinks Industry | AG Barr | News Media | Financial Times (FT.Com) |
| Irn Bru tells City it's strong enough to bear sugar levy. | 30 | 3 | 2016 | Drinks Industry | AG Barr | News Media | i |
| The argument against sugar tax made simple.. | 30 | 3 | 2016 | Drinks Industry | Coca-Cola enterprises | Trade | Just-Drinks |
| Irn-Bru and Tizer-maker AG Barr posts hike in profits and demands urgent meeting with the Treasury over sugar tax. | 30 | 3 | 2016 | Drinks Industry | AG Barr | News Media | Mail Online |
| Barr's not bitter about sugar tax. | 30 | 3 | 2016 | Drinks Industry | AG Barr | News Media | The Daily Express |
| Sweet relief from sugar tax. | 30 | 3 | 2016 | Drinks Industry | AG Barr | News Media | The Daily Telegraph |
| AG Barr to cut sugar in drinks before UK levy. | 30 | 3 | 2016 | Drinks Industry | AG Barr | News Media | The Irish Times |
| Irn-Bru maker sheds the sugar to beat fizzy drinks tax. | 30 | 3 | 2016 | Drinks Industry | AG Barr | News Media | The Times |
| What will a UK sugar tax mean for soft drinks producers? - Comment. | 31 | 3 | 2016 | Food Industry | Danone | Trade | Just-Drinks |
| Britain will be biggest nanny state in the EU thanks to sugar tax, experts say. | 31 | 3 | 2016 | Think-Tank | Institute of economic affairs | News Media | thesun.co.uk |
| Sugar tax is ‘a slippery slope’. | 1 | 4 | 2016 | Association | Food and drink federation, Institute of economic affairs | Trade | British Baker |
| Sugar soft drinks tax: a ‘dead cat’ distraction. | 1 | 4 | 2016 | Drinks Industry | SOURCE- Anonymous | Trade | Food Manufacture |
| What happened next George Osborne's sugar tax. | 2 | 4 | 2016 | Drinks Industry | Coca-Cola | News Media | Telegraph Magazine |
| Irn-Bru owner AG Barr brushes off worries about the sugar tax. | 2 | 4 | 2016 | Drinks Industry | AG Barr | Trade | The Grocer |
| Which supermarket own-brand fizzy drinks will be hit hardest by the sugar tax? | 3 | 4 | 2016 | Food Industry | Waitrose | News Media | The Telegraph Online |
| Waitrose faces biggest sugar tax hit for own-brand drinks. | 4 | 4 | 2016 | Food Industry | Waitrose | News Media | The Daily Telegraph |
| ‘Cereals should be hit by sugar tax’. | 6 | 4 | 2016 | Food Industry | Naturo Whole Wheat Grain | News Media | Carmarthen Journal |
| Letter: Cereals next for sugar tax? | 7 | 4 | 2016 | Citizen | People against sugar tax | News Media | Barry And District News |
| Jamie Oliver 'expects kicking' over sugar tax. | 12 | 4 | 2016 | Food Industry | Wetherspoons | News Media | The Guardian |
| Ministers urged to introduce sugar tax in childhood obesity report. | 12 | 4 | 2016 | Association | Food and drink federation | News Media | The Guardian |
| David Cameron faces pressure to back sugar tax. | 12 | 4 | 2016 | Association | Food and drink federation, Cadbury's | News Media | The Guardian |
| Boss of British Sugar voices unease at Government tax plans for soft drinks. | 19 | 4 | 2016 | Association | Associated British foods | News Media | Bury Free Press |
| ABF hits out at sugar tax while Primark suffers first fall in 12 years. | 19 | 4 | 2016 | Association | Associated British foods | News Media | The Telegraph Online |
| Sugar chief blasts Tories' fat tax. | 20 | 4 | 2016 | Association | Associated British foods | News Media | Daily Record |
| Sugar tax will have no effect, says ABF chief as Primark falters. | 20 | 4 | 2016 | Association | Associated British foods | News Media | The Daily Telegraph |
| From Zero to hero: Coca-Cola’s sugar-free drink to taste more like original Coke — in bid to beat sugar tax. | 20 | 4 | 2016 | Drinks Industry | Coca-Cola | News Media | thesun.co.uk |
| Soft-drinks firms weigh up sugar-tax response. | 1 | 5 | 2016 | Drinks Industry | Britvic | Trade | Marketing |
| Sugar tax is 'best way' to beat obesity. | 1 | 5 | 2016 | Association | British soft drinks association | News Media | The Sunday Times |
| Irn-Bru maker plays down financial impact of sugar tax. | 6 | 5 | 2016 | Drinks Industry | AG Barr | News Media | The Guardian |
| Unilever boss warns UK against sugar tax. | 6 | 5 | 2016 | Food Industry | Unilever | News Media | The Guardian |
| Sugar tax leaves a bitter taste in mouth. | 12 | 5 | 2016 | Citizen | People against sugar tax | News Media | Bath Chronicle |
| George Osborne's 'discriminatory' sugar tax looks set to fall foul of EU laws. | 16 | 5 | 2016 | Association | British soft drinks association Coca-Cola | News Media | thesun.co.uk |
| Britvic profits increase sweetens pill of looming sugar tax. | 19 | 5 | 2016 | Drinks Industry | Britvic | News Media | Financial Times (FT.Com) |
| Drinks giant ready for sugar tax as it unveils rise in profits. | 20 | 5 | 2016 | Drinks Industry | Britvic | News Media | Eastern Daily Press |
| Britvic profits up as firm prepares for the sugar tax. | 20 | 5 | 2016 | Drinks Industry | Britvic | News Media | Evening News (Norwich) |
| Sugar tax spurs Britvic to change drinks recipes by 2020. | 20 | 5 | 2016 | Drinks Industry | Britvic, Coca-Cola, Mars foods | News Media | Independent Online |
| Britvic to change recipes for more of its drinks to avoid sugar tax. | 20 | 5 | 2016 | Drinks Industry | Britvic | News Media | The Guardian |
| Britvic optimistic on sugar tax after drinks shake-up. | 20 | 5 | 2016 | Drinks Industry | Britvic | News Media | The Times |
| Pepsi recipe to be changed in response to sugar tax. | 21 | 5 | 2016 | Drinks Industry | Britvic, Coca-Cola, Mars foods | News Media | The Independent |
| Sham of sugar tax as sales plummet. | 21 | 5 | 2016 | Drinks Industry | Coca-Cola | News Media | The Sun |
| Chocs away: Calls for sugar tax to be scrapped as Government reveals it could be EXTENDED to include sweets, milkshakes and even breakfast cereals. | 24 | 5 | 2016 | Association | Food and drink federation | News Media | thesun.co.uk |
| The Coca-Cola Co takes aim at UK sugar tax. | 26 | 5 | 2016 | Drinks Industry | Coca-Cola | Trade | Just-Drinks |
| We're tackling the sugar question but tax isn't the answer. | 26 | 5 | 2016 | Drinks Industry | Coca-Cola | News Media | The Daily Telegraph |
| Coke boss condemns Government sugar tax. | 26 | 5 | 2016 | Drinks Industry | Coca-Cola | News Media | The Daily Telegraph |
| Coke are furious at sugar tax proposal. | 27 | 5 | 2016 | Drinks Industry | Coca-Cola | News Media | The Sun |
| TaxPayers' Alliance raps sugar levy as unfair attack on the poor. | 30 | 5 | 2016 | Citizen | Tax payers alliance | News Media | Belfast Telegraph |
| Demand for sugar tax axe. | 30 | 5 | 2016 | Citizen | Tax payers alliance | News Media | Daily Star |
| Sugar tax will be an arbitrary penalty that hits the poor hardest, consumer group warns. | 30 | 5 | 2016 | Citizen | Tax payers alliance | News Media | Independent Online |
| Super-sweet drinks that won't be hit by sugar tax: New laws branded a farce after range of shakes are exempt because they contain milk. | 30 | 5 | 2016 | Citizen | Tax payers alliance | News Media | Mail Online |
| Sugar tax 'exempts the middle classes'. | 30 | 5 | 2016 | Citizen | Tax payers alliance | News Media | The Daily Telegraph |
| Critics of sugar tax highlight anomalies on drinks. | 30 | 5 | 2016 | Citizen | Tax payers alliance | News Media | The Herald |
| 'Hard-up worst hit by a tax on sugar'. | 30 | 5 | 2016 | Citizen | Tax payers alliance | News Media | The Sun |
| Middle-class latte drinkers will escape the sugar tax, while poor will pay. | 30 | 5 | 2016 | Citizen | Tax payers alliance | News Media | The Telegraph Online |
| Sugar tax on fizzy drinks will unfairly hit the poor, critics warn. | 30 | 5 | 2016 | Citizen | Tax payers alliance | News Media | thesun.co.uk |
| Sugar tax is destined to fail, claims legal expert. | 1 | 6 | 2016 | Other | DWF | Trade | Food Manufacture |
| Sugar tax benefits. | 2 | 6 | 2016 | Citizen | Tax payers alliance | News Media | The Daily Telegraph |
| The critics have got it wrong? a sugar tax will hit the sweet spot. | 2 | 6 | 2016 | Think-Tank | Institute of economic affairs | News Media | thetimes.co.uk |
| Pitfalls of a sugar tax. | 4 | 6 | 2016 | Citizen | Tax payers alliance | News Media | The Daily Telegraph |
| 09:32 EDT Coca-Cola to collaborate with U.K., won't sue over sugar tax, FT... | 6 | 6 | 2016 | Drinks Industry | Coca-Cola | Trade | Theflyonthewall.com |
| Coca-Cola cans talk of fight against sugar tax; Interview: Sol Daurella. | 7 | 6 | 2016 | Drinks Industry | Coca-Cola european partners | News Media | Financial Times |
| Sugar prices soar as tax war rumbles on. | 10 | 6 | 2016 | Citizen | Tax payers alliance | Trade | British Baker |
| Evidence from Mexico proves UK's sugar tax is not so sweet. | 13 | 6 | 2016 | Citizen | Tax payers alliance | News Media | City AM |
| Tax credits sweeten 'sugar levy' for soft drinks producers; R&D relief. | 24 | 6 | 2016 | Association | British soft drinks association, Food and drink federation | News Media | Financial Times |
| Cameron refuses to rule out sugar tax on drinks. | 24 | 6 | 2016 | Association | British soft drinks association | News Media | The Guardian |
| Sugar tax won’t cut obesity. | 7 | 7 | 2016 | Citizen | People against sugar tax | News Media | Tamworth Herald |
| Coca-Cola boss claims sugar tax is bad for consumers and will act as a distraction from efforts to switch to lower calorie products. | 10 | 7 | 2016 | Drinks Industry | Coca-Cola, Food and drink federation | News Media | Mail Online |
| UK Food & Drink Federation seeks sugar tax pause. | 11 | 7 | 2016 | Association | Food and drink federation | Trade | Just-Drinks |
| UK sugar tax will hit jobs and economy, say campaigners. | 11 | 7 | 2016 | Citizen | Tax payers alliance | Trade | Just-Food |
| Sugar tax 'will not cut obesity but will harm UK economy'. | 11 | 7 | 2016 | Citizen | Tax payers alliance, Coca-Cola | News Media | The Daily Telegraph |
| Halt sugar tax introduction, urges food and drink industry. | 11 | 7 | 2016 | Association | Food and drink federation, Coca-Cola | News Media | The Guardian |
| Food and drink industry calls for sugar tax delay after Brexit uncertainty. | 12 | 7 | 2016 | Drinks Industry | Food and drink federation, Coca-Cola | News Media | The Independent |
| This week in soft drinks & bottled water, featuring the potential in hangovers, a new sugar tax in South Africa and The Coca-Cola Co's Olympic campaign.. | 15 | 7 | 2016 | Drinks Industry | PepsiCo, Lucozade Ribena Suntory | Trade | Just-Drinks |
| Sugar tax pitfalls. | 18 | 7 | 2016 | Citizen | People against sugar tax | News Media | The Daily Telegraph |
| Barr promises new Irn-Bru swaps sugar for flavour after tax changes. | 21 | 7 | 2016 | Drinks Industry | AG Barr | News Media | The Herald |
| 4k jobs threat of sugar tax. | 8 | 8 | 2016 | Association | Oxford economics, British soft drinks association | News Media | The Sun |
| bittersweet George Osborne’s sugar tax on soft drinks set to put 4,000 British jobs at risk. | 8 | 8 | 2016 | Association | Oxford economics, British soft drinks association | News Media | thesun.co.uk |
| Sugar tax will hit jobs and cost UK economy. | 9 | 8 | 2016 | Citizen | Oxford economics, Tax payers alliance | News Media | City AM |
| Economic concerns over sugar tax. | 10 | 8 | 2016 | Association | Oxford Economics British soft drinks association | News Media | Eastern Daily Press |
| Sugar tax. | 11 | 8 | 2016 | Citizen | People against sugar tax | News Media | Knutsford Guardian |
| Sugar tax risks 4,000 jobs - UK soft drinks industry. | 12 | 8 | 2016 | Association | British soft drinks association, Oxford EconomicS, Food and drink federation | Trade | Just-Drinks |
| This week in soft drinks & bottled water featuring Monster's global ambitions, the future of bottled water and UK sugar tax latest. | 12 | 8 | 2016 | Drinks Industry | Monster beverage corp, Coca-Cola, Refresco Group, PepsiCo, Fever-Tree | Trade | Just-Drinks |
| A SUGAR tax on soft [...]. | 16 | 8 | 2016 | Association | Oxford Economics, British soft drinks association | News Media | Daily Record |
| Sugar tax on drinks ‘will save NHS cash’. | 16 | 8 | 2016 | Association | Oxford economics, British soft drinks association | News Media | Evening Times |
| Do you agree with sugar tax on drinks? Four in ten want to add it to CHOCOLATE too. | 16 | 8 | 2016 | Association | Oxford economics, British soft drinks association | News Media | express.co.uk |
| Businesses sweet on sugar launch 'can the tax' campaign; Soft drinks. | 16 | 8 | 2016 | Citizen | Face the facts, can the tax, British soft drinks association, Oxford economics | News Media | Financial Times |
| UK businesses unite to fight sugar tax. | 16 | 8 | 2016 | Citizen | Face the facts, can the tax, British soft drinks association, Oxford economics, Scottish grocers federation, Automatic vending association, Scottish licensed trade association | News Media | Financial Times (FT.Com) |
| Report warns of 4,000 job losses due to sugar tax on drinks. | 16 | 8 | 2016 | Drinks Industry | Oxford Economics, British soft drinks association, Britvic | News Media | Huddersfield Examiner |
| Sugar tax: UK businesses claim levy will result in job losses and higher prices. | 16 | 8 | 2016 | Drinks Industry | Face the facts, can the tax, British soft drinks association, Coca-Cola, Oxford economics, British beer and pub association, Britvic | News Media | Independent Online |
| Sugar tax could destroy thousands of jobs. | 16 | 8 | 2016 | Association | Oxford economics, British soft drinks association | News Media | Oxford Mail |
| Sugar tax 'will cut just 5 calories'. | 16 | 8 | 2016 | Association | Oxford economics, British soft drinks association | News Media | Scottish Daily Mail |
| British Sugar joins campaign against planned soft drinks tax. | 16 | 8 | 2016 | Association | Associated British foods, British sugar, British soft drinks association, the British beer and pub association, Federation of wholesale distributors, Oxford economics, Automatic vending association | News Media | The Daily Telegraph |
| Sugar tax's industry opponents team up before consultation. | 16 | 8 | 2016 | Citizen | Face the facts, can the tax, Oxford economics, British soft drinks association, British beer and pub association, Coca-Cola, Food and drink federation | News Media | The Guardian |
| Warning that sugar tax on soft drinks could lead to 4,000 job losses. | 16 | 8 | 2016 | Drinks Industry | Oxford Economics, British soft drinks association, Britvic, AG Barr | News Media | The Herald |
| Job fears mount as businesses unite to fight UK sugar tax. | 16 | 8 | 2016 | Association | Associated British foods, British sugar, British soft drinks association, the British beer and pub association, Federation of wholesale distributors, Oxford economics, National federation of retail newsagents | News Media | The Telegraph Online |
| 'Sugar tax puts 4,000 jobs at risk'. | 16 | 8 | 2016 | Association | Oxford Economics British soft drinks association | News Media | The Western Mail |
| fight the fat Plans for £1 billion sugar tax on soft drinks to be launched this week. | 16 | 8 | 2016 | Association | Face the facts, can the tax, British soft drinks association | News Media | thesun.co.uk |
| Sugar tax will be bad news for economy. | 17 | 8 | 2016 | Citizen | People against sugar tax | News Media | Bournemouth Echo |
| Sugar tax 'will cut just five calories': Soft drinks industry says health benefit to consumers equates to a bite of an apple a day - but could cost 4,000 jobs. | 17 | 8 | 2016 | Association | Oxford economics, British soft drinks association | News Media | Mail Online |
| Britain launches soft drinks sugar tax to fight obesity. | 18 | 8 | 2016 | Association | British soft drinks association | Trade | Reuters Health E-Line |
| Britain seeks to fight the fat with soft drinks sugar levy. | 18 | 8 | 2016 | Association | British soft drinks association | News Media | Reuters News |
| Is a sugar tax enough to tackle childhood obesity? - live debate. | 18 | 8 | 2016 | Association | British soft drinks association | News Media | The Guardian |
| Government moves on sugar tax are 'feeble', health campaigners say. | 18 | 8 | 2016 | Association | Face the facts, can the tax, British soft drinks association | News Media | The Independent |
| Government admits it hasn't yet determined sugar levy cut-off. | 20 | 8 | 2016 | Think-Tank | Oxford economics | Trade | The Grocer |
| 'Face the Facts, Can the Tax' campaign fights sugar levy. | 20 | 8 | 2016 | Citizen | Face the facts, can the tax, Oxford economics, British soft drinks association, FWD | Trade | The Grocer |
| BURST HIS TAX BUBBLE Government backtrack on George Osborne’s sugar tax plan after muddle in report which says producers should pay. | 21 | 8 | 2016 | Association | British soft drinks association | News Media | thesun.co.uk |
| The sugar tax won't beat obesity and should be binned. | 22 | 8 | 2016 | Think-Tank | Institute of economic affairs | News Media | The Times |
| £1bn TAX POP AT POOR Sugar tax just a way of getting money out of the poor, claims think tank. | 22 | 8 | 2016 | Think-Tank | Institute of economic affairs | News Media | thesun.co.uk |
| Sugar tax sets a dangerous precedent. | 27 | 8 | 2016 | Association | British soft drinks association | Trade | The Grocer |
| London South Bank University - LSBU set to host sugar tax debate. | 1 | 9 | 2016 | Other | DWF, People against sugar tax | News Media | ENP Newswire |
| This week in soft drinks & bottled water, featuring the sugar tax battle in the US, brand Cocozia goes up for sale and Sodastream's new home-carbonation machine. | 16 | 9 | 2016 | Association | American beverage association | Trade | Just-Drinks |
| Calls for sugar tax to be dropped as children's intake falls. | 17 | 9 | 2016 | Association | British soft drinks association, Food and drink federation | Trade | The Grocer |
| Irn-Bru maker tackles tax hike with sugar-free push. | 27 | 9 | 2016 | Drinks Industry | AG Barr | News Media | London Evening Standard |
| Irn-Bru maker AG Barr tackles tax hike with sugar-free push. | 27 | 9 | 2016 | Drinks Industry | AG Barr | News Media | London Evening Standard Online |
| Irn Bru maker AG Barr rails against ‘punitive’ sugar tax as sales fall. | 27 | 9 | 2016 | Drinks Industry | AG Barr | News Media | The Telegraph Online |
| Irn-Bru maker's profits flattened by sugar tax. | 28 | 9 | 2016 | Drinks Industry | AG Barr | News Media | The Daily Telegraph |
| SOFT DRINKS BLOW Irn Bru to slash 10% of its workforce over Government’s ‘sugar tax’. | 28 | 9 | 2016 | Drinks Industry | AG Barr | News Media | thesun.co.uk |
| FAKE FIZZ FURORE Dangerous boom in counterfeit pop ‘will threaten UK once sugar tax comes into force’. | 28 | 9 | 2016 | Think-Tank | Institute of economic affairs | News Media | thesun.co.uk |
| Investors Chronicle - magazine and web content: Drinks makers' anger over proposed sugar levy flares. | 29 | 9 | 2016 | Drinks Industry | AG Barr, Britvic. Nichols | Trade | Investors Chronicle - Magazine and Web Content |
| Labour's Luciana Berger slams plans for sugar levy. | 1 | 10 | 2016 | Association | British soft drinks association | Trade | The Grocer |
| Soft drinks maker in Bottesford warns of harmful impact of sugar tax plan. | 10 | 10 | 2016 | Food Industry | Belvoir fruit farms | News Media | Grantham Journal |
| can sugar tax Theresa May urged to scrap Sugar Tax amid claims cutting portions is TEN TIMES more effective at fighting flab. | 13 | 10 | 2016 | Association | British soft drinks association | News Media | thesun.co.uk |
| A lot of fizz: Sugar tax could fuel illicit soft drinks trade. | 14 | 10 | 2016 | Association | Face the facts, can the tax, British soft drinks association | News Media | City AM |
| Milkshakes should not be exempt from sugar tax says BSDA. | 15 | 10 | 2016 | Association | British soft drinks association | Trade | The Grocer |
| Health chiefs call for sugar tax to hit coffee chains. | 16 | 10 | 2016 | Association | British soft drinks association | News Media | The Sunday Times |
| Letter to the Editor: Scrap the sugar tax, people don't want it. | 19 | 10 | 2016 | Citizen | People against sugar tax | News Media | Bournemouth Echo |
| Portugal unveils plans for a sugar tax on soft drinks. | 22 | 10 | 2016 | Association | British soft drinks association | Trade | The Grocer |
| Lucozade and Ribena are to reduce their sugar content by 50% - avoiding the Government's sugar tax. | 9 | 11 | 2016 | Drinks Industry | Lucozade Ribena Suntory | News Media | Mail Online |
| Sugar in Lucozade reduced by 50% to escape tax penalty. | 9 | 11 | 2016 | Drinks Industry | Lucozade Ribena Suntory, Tesco | News Media | thetimes.co.uk |
| UK food body's member brands don't care about the sugar tax, claims children campaign. | 2 | 12 | 2016 | Association | Food and drinks federation | News Media | City AM |
| Soft drinks sugar levy 'divides industry', survey shows. | 3 | 12 | 2016 | Association | Food and drinks federation | Trade | The Grocer |
| LETTER: No need for a sugar tax. | 4 | 12 | 2016 | Citizen | People against sugar tax | News Media | Somerset County Gazette |
| Is the government about to publish plans for a sugar tax?. | 5 | 12 | 2016 | Association | British soft drinks association, Tax payers alliance | News Media | Dorset Echo |
| Drinks companies trim exposure to limber up for sugar levy; Anti-obesity initiative. | 5 | 12 | 2016 | Drinks Industry | Coca-Cola, Lucozade Ribena Suntory, Britvic, AG Barr, Tesco, British soft drinks association | News Media | Financial Times |
| UK Treasury postpones finer details on sugar tax charges.. | 5 | 12 | 2016 | Drinks Industry | Lucozade Ribena Suntory, Coca-Cola | Trade | Just-Drinks |
| UPDATE 1-Britain publishes draft sugar tax. | 5 | 12 | 2016 | Association | British soft drinks association | News Media | Reuters News |
| Britain publishes draft sugar tax. | 5 | 12 | 2016 | Drinks Industry | Coca-Cola, PepsiCo | News Media | Reuters News |
| Sugar tax could mean rise in general taxation, pressure group claims. | 5 | 12 | 2016 | Citizen | Tax payers alliance | News Media | The Telegraph Online |
| not so sweet Sugar tax could lead to struggling British families paying even HIGHER taxes, claims new report. | 5 | 12 | 2016 | Citizen | Tax payers alliance | News Media | thesun.co.uk |
| UK Government sets out plans for sugar tax on soft drinks. | 6 | 12 | 2016 | Association | British soft drinks association | Trade | FinancialWire |
| Sugar tax to raise prices of favourite fizzy drinks. | 6 | 12 | 2016 | Citizen | British soft drinks association, Tax payers alliance | News Media | i |
| Treasury vows that the 'sugar tax' will go ahead as planned. | 6 | 12 | 2016 | Drinks Industry | Britvic, British soft drinks association, Tax payers alliance | News Media | Independent Online |
| Sugar tax is unfair to blackcurrant drinks, says Ribena boss. | 6 | 12 | 2016 | Drinks Industry | Lucozade Ribena Suntory | News Media | The Telegraph Online |
| Firms cut sugar before tax bites. | 6 | 12 | 2016 | Drinks Industry | Lucozade Ribena Suntory, Tesco | News Media | The Times |
| From mandatory exercise to 'nannying' sugar tax: Theresa May's war on FAT BRITAIN revealed. | 10 | 12 | 2016 | Citizen | Tax payers alliance | News Media | dailystar.co.uk |
| What now for the soft drinks sugar levy?. | 10 | 12 | 2016 | Drinks Industry | British soft drinks association, Lucozade Ribena Suntory, | Trade | The Grocer |
| UK tax on sugar aims to reduce number of obese children by 7%. | 16 | 12 | 2016 | Association | British soft drinks association | News Media | Financial Times (FT.Com) |
| 'Health by stealth' sugar tax could slash rates of childhood obesity by 10 per cent. | 16 | 12 | 2016 | Drinks Industry | Lucozade Ribena Suntory | News Media | The Telegraph Online |
| Sugar tax 'will stop 10% of child obesity'. | 16 | 12 | 2016 | Drinks Industry | Lucozade Ribena Suntory, Tesco | News Media | The Times |
| Mexico's sugar tax leads to fall in consumption for second year running. | 23 | 2 | 2017 | Association | British soft drinks association | News Media | The Guardian |
| Sugar levy set to raise £415m to support schools' fight against obesity. | 28 | 2 | 2017 | Association | British soft drinks association | News Media | The Telegraph Online |
| AG Barr to take 90% of portfolio out of UK sugar tax reach.. | 1 | 3 | 2017 | Drinks Industry | AG Barr | Trade | Just-Drinks |
| Soft drinks giants ramp up sugar efforts as levy looms. | 4 | 3 | 2017 | Drinks Industry | AG Barr | Trade | The Grocer |
| Budget 2017: New sugar tax confirmed by Philip Hammond in fight to combat rising obesity. | 8 | 3 | 2017 | Association | British soft drinks association | News Media | Independent Online |
| Sugar tax to fight rising obesity levels. | 9 | 3 | 2017 | Association | British soft drinks association | News Media | Belfast Telegraph |
| Sweet or sour: The sugar levy will make less than expected. | 9 | 3 | 2017 | Association | Food and drinks federation | News Media | City AM |
| School sport funding on track despite drop in sugar revenues; Soft drinks tax. | 9 | 3 | 2017 | Drinks Industry | Coca-Cola european partners | News Media | Financial Times |
| Budget 2017: Revenues from UK’s incoming sugar tax to fall short. | 9 | 3 | 2017 | Drinks Industry | AG Barr, Lucozade Ribena Suntory, Coca-Cola european partners, British soft drinks association | News Media | Financial Times (FT.Com) |
| Sugar tax that will add 8p to a can of Coca-Cola WILL go ahead next year, Hammond confirms. | 9 | 3 | 2017 | Association | British soft drinks association | News Media | Mail Online |
| 'Sugar tax' to deliver £500m less to Treasury. | 9 | 3 | 2017 | Drinks Industry | AG Barr | News Media | Scottish Daily Mail |
| Sugar tax yield to fall after drink makers change recipes. | 9 | 3 | 2017 | Drinks Industry | AG Barr | News Media | The Daily Telegraph |
| Sugar tax and calorie limits hailed as solution to obesity. | 9 | 3 | 2017 | Drinks Industry | AG Barr, Food and drink federation | News Media | The Herald |
| Nix & Kix reduces sugar content ahead of UK sugar tax.. | 16 | 3 | 2017 | Drinks Industry | Nix&Kix | Trade | Just-Drinks |
| Coke's 'plot on sugar tax'. | 27 | 3 | 2017 | Drinks Industry | Coca-Cola | News Media | The Daily Mirror |
| CAN IT What is the Coca-Cola recipe, how will the sugar tax affect the price of Coke and how much sugar is in a can of Coke?. | 27 | 3 | 2017 | Drinks Industry | Coca-Cola | News Media | thescottishsun.co.uk |
| CAN IT What is the Coca-Cola recipe, how will the sugar tax affect the price of Coke and how much sugar is in a can of Coke?. | 27 | 3 | 2017 | Drinks Industry | Coca-Cola | News Media | thesun.co.uk |
| Sugar tax hard to swallow, says boss of AG Barr. | 28 | 3 | 2017 | Drinks Industry | AG Barr | News Media | London Evening Standard |
| LIFTING THE LID Coca-Cola ‘fighting secret battle against sugar tax’ by meeting with ministers and funding health studies, according to leaked emails detailed on new documentary. | 28 | 3 | 2017 | Drinks Industry | Coca-Cola | News Media | thesun.co.uk |
| Putting sugar tax on ice during Brexit talks would be 'well advised'. | 4 | 4 | 2017 | Association | Food and drinks federation | News Media | The Telegraph Online |
| Put sugar tax back until after Brexit, says soft drinks trade. | 5 | 4 | 2017 | Association | Food and drinks federation | News Media | The Daily Telegraph |
| SHS Drinks takes Shloer under UK sugar tax threshold.. | 19 | 4 | 2017 | Drinks Industry | SHS Drinks | Trade | Just-Drinks |
| Coca-Cola could face £200m sugar tax from Classic alone. | 22 | 4 | 2017 | Drinks Industry | Coca-Cola european partners | Trade | The Grocer |
| FIZZY POP TAX GREEN LIGHT Sugar tax will be introduced next April after Parliament approves controversial price hike. | 26 | 4 | 2017 | Citizen | Tax payers alliance | News Media | thesun.co.uk |
| Sugar tax must apply to sweets as well as drinks, say campaigners. | 12 | 5 | 2017 | Association | Food and drinks federation | News Media | The Guardian |
| Lucozade Ribena Suntory cuts sugar to avoid tax charge. | 1 | 6 | 2017 | Drinks Industry | Lucozade Ribena Suntory | Trade | Food Manufacture |
| 'Sugar tax should be extended to hit retailers harder' - FSS. | 24 | 6 | 2017 | Association | BRC, Food and drink federation, Advertising association, British soft drinks association | Trade | The Grocer |
| Look beyond the sugar tax to see Britvic's true potential as a major global player QuestorTrust Bargains QuestorIncome Portfolio. | 9 | 7 | 2017 | Drinks Industry | Britvic | News Media | The Sunday Telegraph |
| Vimto maker to avoid sugar levy. | 21 | 7 | 2017 | Drinks Industry | Nichols | News Media | The Daily Telegraph |
| Vimto maker's brands won't trigger sugar tax after focus on new recipes. | 21 | 7 | 2017 | Drinks Industry | Nichols | News Media | The Telegraph Online |
| Coke, Monster to be only brands left in UK sugar tax firing line - Coca-Cola European Partners.. | 11 | 8 | 2017 | Drinks Industry | Coca-Cola european partners | Trade | Just-Drinks |
| How taxes on sugar work around the world. | 30 | 9 | 2017 | Association | Food and drinks federation | Trade | The Grocer |
| Sugar tax costs us all. | 6 | 10 | 2017 | Citizen | People against sugar tax | News Media | Wiltshire Times |
| Dismay as drinks firm axes Moray Cup due to sugar tax production. | 26 | 10 | 2017 | Drinks Industry | Cott Beverages | News Media | Evening Express |
| Lucozade sales plummet after brand dramatically cuts amount of sugar in drinks following tax levy. | 5 | 11 | 2017 | Drinks Industry | Lucozade Ribena Suntory | News Media | irishmirror.ie |
| Revealed, 'threat' by Coca-Cola to cut investment over sugar tax. | 5 | 11 | 2017 | Drinks Industry | Coca-Cola, Food and drink federation | News Media | The Sunday Telegraph |
| Revealed: 'Threat' by Coca-Cola to cut investment over sugar tax. | 5 | 11 | 2017 | Association | Coca-Cola, Food and drink federation | News Media | The Telegraph Online |
| Barr ups defences against sugar tax. | 8 | 11 | 2017 | Drinks Industry | AG Barr | News Media | The Herald |
| Britvic shoots higher — but sugar tax sours outlook. | 29 | 11 | 2017 | Drinks Industry | Britvic | News Media | London Evening Standard |
| Britvic shoots higher despite looming sugar tax. | 29 | 11 | 2017 | Drinks Industry | Britvic | News Media | London Evening Standard Online |
| UPDATE 2-Britvic core earnings rise; says well placed to take on sugar tax. | 29 | 11 | 2017 | Drinks Industry | Britvic | News Media | Reuters News |
| Britvic warns of uncertainty from sugar tax. | 29 | 11 | 2017 | Drinks Industry | Britvic | News Media | The Telegraph Online |
| Overhaul costs take fizz out of Britvic profits as sugar tax looms. | 30 | 11 | 2017 | Drinks Industry | Britvic | News Media | The Daily Telegraph |
| No feeling sour over sugar tax. | 30 | 11 | 2017 | Drinks Industry | Britvic | News Media | The Times |
| Innovations bear fruit for Ribena as drinks market faces sugar levy. | 27 | 12 | 2017 | Drinks Industry | Lucozade Ribena Suntory | News Media | i |
| Shoppers 'panic buy' Irn Bru ahead of recipe changing to avoid sugar tax. | 4 | 1 | 2018 | Drinks Industry | AG Barr | News Media | London Evening Standard Online |
| Irn-Bru fans in a panic as 117-year-old recipe is changed to beat sugar tax. | 5 | 1 | 2018 | Drinks Industry | AG Barr | News Media | i |
| Fans rush to stockpile Irn Bru before makers of the traditional Scottish drink halve sugar content to beat new tax. | 5 | 1 | 2018 | Drinks Industry | AG Barr | News Media | Mail Online |
| NATIONAL TREASURE Hands off our Irn Bru petition signed by over 10,000 Scots after AG Barr reveal recipe will be changing FOR GOOD within days due to sugar tax. | 5 | 1 | 2018 | Drinks Industry | AG Barr | News Media | thescottishsun.co.uk |
| Coca-Cola will sell smaller bottles at higher prices rather than alter famous recipe in response to the sugar tax. | 6 | 1 | 2018 | Drinks Industry | Coca-Cola | News Media | Mail Online |
| COKE SHRINKS Coca-Cola to make its bottles SMALLER while hiking prices in bid to beat sugar tax. | 6 | 1 | 2018 | Drinks Industry | Coca-Cola | News Media | thescottishsun.co.uk |
| 11 things you said about Coca-Cola's plans to raise prices in response to sugar tax. | 10 | 1 | 2018 | Drinks Industry | Coca-Cola, AG Barr | News Media | The Sunderland Echo |
| Coca-Cola to shrink bottles and raise prices due to UK sugar tax. | 14 | 1 | 2018 | Drinks Industry | Coca-Cola | News Media | Milton Keynes Citizen |
| Coca-Cola blames sugar tax as it cuts coke bottle size and puts prices up. | 16 | 1 | 2018 | Drinks Industry | Coca-Cola european partners, AG Barr | News Media | Independent Online |
| Coca-Cola to cut bottle size and hike prices in response to sugar tax. | 16 | 1 | 2018 | Drinks Industry | Coca-Cola european partners | News Media | London Evening Standard Online |
| IN A FIZZ Coca-Cola to cut bottle size but put up prices in bid to beat sugar tax. | 16 | 1 | 2018 | Drinks Industry | Coca-Cola | News Media | thescottishsun.co.uk |
| Sugar tax sours Wetherspoon forecast. | 25 | 1 | 2018 | Food Industry | Wetherspoons | News Media | The Times |
| Supermarket deals on chocolate 'sharing' packs should be banned - the campaign calling for a fresh 20% sugar tax. | 26 | 1 | 2018 | Association | Food and drinks federation | News Media | Mirror.co.uk |
| Coca-Cola launches three new drinks ahead of UK sugar tax. | 29 | 1 | 2018 | Drinks Industry | Coca-Cola | News Media | The Telegraph Online |
| Strong demand for fizzy drinks lifts UK-based Britvic as the industry braces for April's 'sugar tax' introduction. | 31 | 1 | 2018 | Drinks Industry | Britvic | News Media | Mail Online |
| AG Barr reveals 99% of portfolio to escape sugar tax; FY 2018 sales to rise - trading update.. | 1 | 2 | 2018 | Drinks Industry | AG Barr | Trade | Just-Drinks |
| Britvic offers helping hand to UK retailers with sugar tax website.. | 8 | 2 | 2018 | Drinks Industry | Britvic | Trade | Just-Drinks |
| Spar zeros in on own-label cola ahead of sugar levy. | 17 | 2 | 2018 | Food Industry | Spar | Trade | The Grocer |
| Sugar tax is a stroke of luck for new venture of former Red Bull chief. | 1 | 3 | 2018 | Drinks Industry | Tenzing | News Media | The Herald |
| Nichols pulls complete soft drinks stable below UK sugar tax line.. | 2 | 3 | 2018 | Drinks Industry | Nichols, AG Barr | Trade | Just-Drinks |
| Cawston Press drops added sugar to beat UK levy, attacks artificial sweeteners.. | 15 | 3 | 2018 | Drinks Industry | Cawston Press | Trade | Just-Drinks |
| Wetherspoon's Tim Martin criticises 'Jamie Oliver's sugar tax' for £3m bill. | 16 | 3 | 2018 | Food Industry | Wetherspoons | News Media | The Telegraph Online |
| Amount raised by Government sugar tax is thought to be £280million LESS than expected as schools fear they will miss out on funding for sport. | 17 | 3 | 2018 | Food Industry | Wetherspoons | News Media | Mail Online |
| JD Wetherspoon chief censures Jamie Oliver over £3m sugar tax. | 17 | 3 | 2018 | Food Industry | Wetherspoons | News Media | The Daily Telegraph |
| Sugar tax ‘will harm pub trade more than grocers’. | 17 | 3 | 2018 | Food Industry | Wetherspoons | News Media | The Herald |
| Sugar tax has sour taste for Wetherspoons. | 17 | 3 | 2018 | Food Industry | Wetherspoons | News Media | The Times |
| SPOONS KNIFE JAMIE Wetherspoons boss blames Jamie Oliver for coming sugar tax price hikes. | 17 | 3 | 2018 | Food Industry | Wetherspoons | News Media | thesun.co.uk |

Supplementary File 3: Full List of actors and the frequency of inclusion in included articles.

| **Actors** | **Frequency** |
| --- | --- |
| Adam Smith Institute | 2 |
| Advertising Association | 2 |
| AG Barr | 60 |
| American Beverage Association | 1 |
| Anonymous Source(s) | 5 |
| Apply Food & Drinks | 1 |
| Associated British foods | 8 |
| Automatic Vending Association | 4 |
| Belvoir Fruit Farms | 1 |
| British Beer and Pub Association | 2 |
| British Beer and Pub Association | 2 |
| British Retail Consortium | 2 |
| British Soft Drinks Association | 74 |
| British Sugar | 2 |
| Britvic | 27 |
| Cadbury's | 1 |
| Cawston Press | 1 |
| CBI | 1 |
| Coca-Cola | 60 |
| Cott Beverages | 1 |
| Danone | 1 |
| DWF | 2 |
| Face the Facts, Can the Tax, | 8 |
| Federation of Wholesale Distributors | 3 |
| Fever-Tree | 1 |
| Food and Drink Federation | 38 |
| Incorporated Society of British Advertisers | 1 |
| Institute of Economic Affairs | 10 |
| Institute of Promotional Marketing, | 1 |
| Lick Creative, | 1 |
| Lucozade Ribena Suntory | 14 |
| Mars Foods | 2 |
| McDonald's | 2 |
| Monster beverage corp, | 1 |
| More Drinks | 1 |
| National Federation of Retail Newsagents | 1 |
| Naturo Whole Wheat Grain | 1 |
| Nichols | 5 |
| Nix & Kix | 1 |
| Oxford Economics | 22 |
| People Against Sugar Tax | 11 |
| PepsiCo | 3 |
| Princes | 1 |
| Refresco Group, | 1 |
| Scottish Grocers Federation | 5 |
| SHS Drinks | 1 |
| Spar | 1 |
| Tax Payers Alliance | 30 |
| Tenzing | 1 |
| Tesco | 5 |
| Unilever | 1 |
| Waitrose | 2 |
| Wetherspoons | 8 |
| **Grand Total** | **443** |
